# Improved Clinical Outcomes in Anastomotic Leak Management Using Preemptive Endoscopic Vacuum Therapy After Esophagectomy

**DOI:** 10.1245/s10434-026-19622-0

**Published:** 2026-04-12

**Authors:** Jennifer Merten, Markus Karl Kostka, Carsten Szardenings, Jens Peter Hölzen, Mazen A. Juratli, Nader El-Sourani, Andreas Pascher, Ann-Kathrin Eichelmann

**Affiliations:** 1 Department of General, Visceral and Transplant Surgery, University Hospital Muenster, Muenster, Germany; 2 Institute of Biostatistics and Clinical Research, University of Muenster, Muenster, Germany

**Keywords:** Esophagectomy, Robot-assisted minimally invasive esophagectomy (RAMIE), Anastomotic leakage, (preemptive) Endoscopic vacuum therapy (pEVT)

## Abstract

**Background:**

Anastomotic leakage (AL) remains one of the most feared complications after esophagectomy. Preemptive endoscopic vacuum therapy (pEVT) has been proposed to support anastomotic healing, but robust clinical data remain limited. This study aimed to evaluate the role of pEVT in the management of AL.

**Methods:**

A retrospective cohort study analyzed 116 esophageal cancer patients who experienced AL after Ivor Lewis esophagectomy between 2012 and 2023. The patients were categorized into two groups: those who received therapeutic EVT (tEVT) after AL diagnosis without prior pEVT and those who experienced AL despite receiving pEVT and subsequently required tEVT as well. Clinical outcomes, leak severity, and hospital metrics were compared with a focus on endoscopic management of AL.

**Results:**

The patients in the pEVT group presented with significantly less severe leaks (ZACC grade II: 36.7% vs. 5.4%; *p* < 0.001), required less intracavitary therapy (43.4% vs. 67.9%; *p* = 0.027), and exhibited lower postoperative inflammation. Preemptive EVT was independently associated with favorable leak grading (odds ratio, 14.4; *p* = 0.004), a 69% reduced need for intracavitary treatment (*p* = 0.027), and markedly shorter intensive care unit (ICU: −74.4%; *p* < 0.001) and hospital (−27.5%; *p* = 0.011) stays. The overall healing rate was high in both groups (pEVT: 85%; non-pEVT: 80.4%).

**Conclusions:**

Preemptive EVT significantly reduces the clinical severity of AL and facilitates faster recovery by shortening ICU and hospital stays. These findings highlight its value as a preventive strategy for high-risk patients undergoing esophagectomy. Prospective studies are needed to validate these promising results.

Esophagectomy is among the most complex procedures in general surgery, associated with a high risk of postoperative complications and considerable morbidity and mortality. Although substantial advances in surgical techniques and perioperative care have been made in recent decades, largely driven by the centralization of services into high-volume centers of excellence, outcomes after esophagectomy remain far from optimal. Despite these improvements, 90-day mortality remains at approximately 4.5%.^1,2^ Given the global incidence of esophageal cancer at 6.3 per 100,000,^3^ the cumulative burden on patients and health care systems is significant.

A particularly feared complication after esophagectomy is anastomotic leakage (AL). This condition can result in life-threatening sequelae such as mediastinitis, sepsis, pleural empyema, erosion of nearby vascular structures such as the aorta, and cardiac complications such as arrhythmias. In addition to the human cost, AL has been shown to substantially increase health care costs and resource utilization, making its prevention a high priority in modern esophageal surgery.^4^

Building on the success of endoscopic vacuum therapy (EVT) in managing AL, its preemptive use (preemptive endoscopic vacuum therapy [pEVT]) has recently emerged as a perioperative strategy to support anastomotic healing.^5^ Early case series suggest improved outcomes after minimally invasive Ivor Lewis esophagectomy, including reduced leakage and zero 30-day mortality in some cohorts.^6^ However, critics argue that patients are exposed to potential drawbacks such as prolonged fasting, the need for parenteral nutrition, and possible interference with enhanced recovery protocols that emphasize early oral intake and mobilization. Taken together, these considerations highlight that current evidence for the routine use of pEVT remains limited and inconclusive.

This study analyzed outcomes of esophageal cancer patients who experienced AL after esophagectomy, comparing those treated with and without pEVT, to assess the impact of pEVT on clinical outcomes. Although we did not assume that pEVT reduces incidence of AL altogether, we hypothesized that it results in a more manageable endoscopic treatment course, marked, for example, by less severe mediastinitis.

## Patients

### Study Population

This retrospective single-center cohort study conducted at a high-volume tertiary care hospital included all patients with esophageal cancer or adenocarcinoma of the gastroesophageal junction (AEG) type I or II tumors who underwent Ivor Lewis esophagectomy with intrathoracic anastomosis between 2012 and June 2023 and subsequently experienced AL. The patients were stratified into two groups based on their EVT approach:

1. Non-preemptive (non-pEVT) group: patients who received therapeutic EVT (tEVT) after the diagnosis of AL
2. pEVT group: patients who received pEVT immediately after surgery (pEVT was introduced at our institution in 2018; from that point onward, all patients received pEVT as part of standard postoperative care) and subsequently experienced AL requiring continuation of treatment with a tEVT approach.

The study was approved by the institutional review board (approval no. 2018-137-f-S) and conducted in accordance with the Declaration of Helsinki. Informed consent was obtained from all patients.

The diagnostic workup included endoscopy with endoscopic ultrasound (EUS), tissue biopsy, cross-sectional imaging, and case discussion by a multidisciplinary tumor board. Neoadjuvant therapy (CROSS or FLOT) was initiated based on current clinical guidelines.^7^ Surgical resection was performed 6 to 8 weeks after completion of neoadjuvant therapy, following previously described techniques.^8,9^

In the early phase of the study period, esophagectomies were primarily performed as open procedures using either hand-sewn or stapled intrathoracic anastomoses. A small number of minimally invasive esophagectomies (MIEs) were performed during the transitional phase. Hybrid robot-assisted minimally invasive esophagectomy (RAMIE) procedures using the da Vinci Si system were performed starting in January 2019, whereas full-RAMIE procedures with the da Vinci Xi system began in January 2021 and subsequently became the standard surgical approach.

Regardless of the surgical access route (open, MIE, or hybrid-RAMIE), all stapled anastomoses were performed using the same standardized technique comprising a circular-stapled, end-to-side intrathoracic esophago-gastrostomy above the azygos vein using either a 25- or 29-mm stapling device. Feeding jejunostomies were selectively placed in patients with preoperative weight loss exceeding 10% of body weight and those requiring essential medications such as neurologic agents or immunosuppressants.

### Study Parameters

Patient records were systematically reviewed for demographic data, comorbidities, oncologic characteristics, surgical details, peri- and postoperative parameters, and outcomes, with a particular focus on the endoscopic management of AL using EVT. To assess AL severity, we applied the Zurich Anastomotic Condition Classification (ZACC), a validated endoscopic grading system based on mucosal ischemia, dehiscence extent, cavity formation, and contamination.^6^

### Application of EVT and Postoperative Management

In both groups, a routine esophagogastroduodenoscopy (EGD) was performed on postoperative day 5 or earlier if clinical signs of anastomotic leakage were present. Preemptive EVT was applied intraoperatively immediately after completion of the anastomosis and before extubation. A customized polyurethane sponge (VivanoMed Foam; Hartmann AG, Heidenheim an der Brenz, Germany) was prepared to fit the anastomotic region, positioned with one third above and two thirds below the anastomosis. Continuous negative pressure of −125 mmHg was applied using a VivanoTec suction device via a transnasally inserted 14 Ch polyvinyl chloride gastroduodenal tube. The sponge was removed during routine EGD on postoperative day 5.

During the initial 5-day pEVT cycle, the patients were kept nil per os. If AL was detected, tEVT and antibiotics (piperacillin/tazobactam) and antifungal therapy (micafungin) were initiated. For cases in which AL led to a mediastinal cavity, the sponge was positioned intracavitarily and exchanged every 3–5 days during follow-up EGD. All EGDs were typically performed under propofol sedation. During tEVT, patients were allowed to drink 200 mL of water per day. Most patients were managed with total parenteral nutrition, whereas some received enteral nutrition through a jejunal feeding tube. For cases in which the AL healed with fistula formation, the resulting anastomotic fistula was primarily managed with barium until complete closure was achieved. All endoscopic procedures were performed by two senior endoscopic surgeons to ensure consistency of technique and assessment.

### Statistical Analyses

Data are presented as frequencies with percentages for categorical variables and as medians with interquartile ranges for continuous variables. Comparisons between the two groups were performed using Fisher’s exact test for categorical variables and the Wilcoxon–Mann–Whitney test for continuous variables. Multivariable regression analyses were performed to evaluate the impact of pEVT on ZACC score (grade II vs. III or IV), the necessity of intracavitary EVT (endoluminal vs. intracavitary), and the duration of overall hospital and intensive care unit (ICU) stay. These models controlled for the following variables: age, sex, American Society of Anesthesiologists (ASA) classification (I/II vs. III/IV), body mass index (BMI), diabetes mellitus, cardiovascular comorbidity, neoadjuvant therapy, presence of a jejunal feeding tube, duration of surgery, and distance of the anastomosis from the incisors. These variables were selected based on a literature review identifying them as related to anastomotic healing.^10–14^ For all regression models, *p* values were derived using the Wald test, and confidence intervals (CIs) were calculated using the normal approximation, following transformation where necessary. All analyses were conducted using R software (version 4.5.0).

## Results

### Baseline Characteristics and Preoperative Findings

The analysis included 116 patients: 56 (48.3%) in the non-pEVT group and 60 (51.7%) in the pEVT group. The median age was 64.5 years, and the median BMI was 27 kg/m^2^. Most patients were male (87.9%). Nearly all the patients were assessed as ASA physical status class 2 or 3, accounting for 114 individuals (98.2%). Regarding other risk factors, 11 patients (9.5%) had diabetes mellitus, and 22 patients (19%) had cardiovascular comorbidities, including peripheral vascular disease. Statistical analysis showed no significant differences between the groups in these baseline characteristics (see Table 1 for details).

**Table 1:** Patient baseline characteristics and preoperative findings

	Total (*n* = 116) *n* (%)	Non-preemptive EVT (*n* = 56) *n* (%)	Preemptive EVT (*n* = 60) *n* (%)	*p* value
Race White	116 (100)	56 (100)	60 (100)	ns
Age: years (range)	64.5 (57–70)	63.5 (55–68)	65.5 (59–71)	ns
Sex				
Male	102 (87.9)	46 (82.1)	56 (93.3)	ns
Female	14 (12.1)	10 (17.9)	4 (6.7)	
ASA classification				
1	1 (0.9)	1 (1.8)	0 (0)	ns
2	57 (49.1)	31 (55.4)	26 (43.3)	
3	57 (49.1)	23 (41.1)	34 (56.7)	
4	1 (0.9)	1 (1.8)	0 (0)	
BMI: kg/m^2^ (range)	26.9 (24.4–31.3)	27.1 (23.4–29.5)	26.8 (24.9–31.8)	ns
Diabetes mellitus	11 (9.5)	8 (14.3)	3 (5)	ns
Cardiovascular comorbidity	22 (19)	11 (19.6)	11 (18.3)	ns
Preoperative findings				
Histology				
Adenocarcinoma	89 (76.7)	40 (71.4)	49 (81.7)	ns
Squamous cell carcinoma	26 (22.4)	15 (26.8)	11 (18.3)	
Other	1 (0.9)	1 (1.8)	0 (0)	
uT				
uT1	10 (8.6)	2 (3.4)	8 (13.3)	ns
uT2	32 (27.6)	16 (28.6)	16 (26.7)	
uT3	72 (62.1)	37 (66.1)	35 (58.3)	
uTx	2 (1.7)	1 (1.8)	1 (1.7)	
N				
N+	87 (75.0)	46 (82.1)	41 (81.7)	ns
Neoadjuvant therapy				
Yes	98 (84.5)	49 (87.5)	49 (81.7)	ns
Chemotherapy	27 (27.6)	13 (26.5)	14 (28.6)	
Radiochemotherapy	71 (72.4)	36 (73.5)	35 (71.4)	
Surgical approach				
Open	50 (43.1)	45 (80.4)	5 (8.3)	**<0.001**
MIE	4 (3.4)	3 (5.4)	1 (1.7)	
Hybrid	14 (12.1)	8 (14.3)	6 (10)	
RAMIE	48 (41.4)	0 (0)	48 (80)	
Anastomotic technique				
Hand-sewn	19 (16.4)	17 (30.4)	2 (3.3)	**<0.001**
Stapler	97 (83.6)	39 (69.6)	58 (96.7)	
Jejunal feeding tube	17 (14.7)	4 (7.1)	13 (21.7)	**0.035**
Duration of surgery: min (range)	359.5 (296.8–438.5)	305 (278.8–360.3)	409.5 (357.8–506)	**<0.001**

Values in bold indicate statistical significance (*p* < 0.05)

EVT, endoscopic vacuum therapy; n, no. of patients; ns, nonsignificant; ASA, American Society of Anesthesiologists; BMI, body mass index; MIE, minimally invasive esophagectomy; RAMIE, robot-assisted minimally invasive esophagectomy

For most of the patients (76.7%), adenocarcinoma was diagnosed. Tumor staging showed that two thirds of the tumors were classified as uT3 (62.1%), followed by uT2 (27.6%) and uT1 (8.6%). Suspected lymph node involvement (uN+) was present in 75% of the cases. A large proportion of the patients (84.5%) received neoadjuvant therapy, and radiochemotherapy was the most commonly administered regimen (72.4%). Open esophagectomy was performed in 43.1% of cases, primarily in earlier years. The MIE, hybrid-RAMIE, and full-RAMIE procedures accounted for 3.4%, 12.1%, and 41.4% of cases, respectively. A significant difference was observed in anastomotic technique: hand-sewn anastomoses were more common in the non-pEVT group (30.4% vs. 3.3%), whereas stapled anastomoses predominated in the pEVT group (69.6% vs. 96.7%) (*p* < 0.001). Furthermore, the duration of surgery was significantly shorter in the non-pEVT group (305 vs. 410 min; *p* < 0.001). A jejunal feeding tube was placed in 14.7% of the patients (see Table 1 for details).

### Therapy Characteristics

Inflammatory markers showed differences between the groups over time. On postoperative day 10, leukocyte counts were significantly higher in the non-pEVT group (13 vs. 10.1; *p* = 0.009), whereas no significant differences were observed on days 3 and 5. Levels of C-reactive protein (CRP) were consistently higher in the non-pEVT group across all measured time points: day 3 (20.2 vs. 15.8 mg/dL; *p* = 0.004), day 5 (16.9 vs. 12.7 mg/dL; *p* = 0.022), and day 10 (19.0 vs. 14.2 mg/dL; *p* = 0.002).

The median time to detection of AL (8 days), distance of anastomosis from incisors (28 cm), and localization of leakage (52% of the AL occurred on the left side) were comparable between the groups. However, the method of AL detection differed significantly (*p* = 0.011). In the pEVT group, AL was more frequently identified during scheduled endoscopic checkups (56.7% vs. 28.6%), whereas in the non-pEVT group, elevated inflammatory markers (44.6% vs. 31.7%), changes in drain characteristics (14.3% vs. 3.3%), and clinical signs or hemodynamic decompensation (12.5% vs. 8.3%) were more common triggers for diagnosis.

Further analysis indicated that patients in the pEVT group tended to present with more manageable defects. Although not statistically significant, three of four patients in the pEVT group (75%) had an anastomotic defect involving less than 25% of the circumference (including defects <10% and 10–25%) compared with 57.2% in the non-pEVT group. The ZACC scores were significantly more favorable in the pEVT group, with a higher proportion of patients exhibiting low-grade (grade 2) leaks (36.7% vs. 5.4%; odds ratio [OR], 10.04; 95% CI 2.73–56.12; *p* < 0.001). Grade 4 leaks were markedly more frequent in the non-pEVT group (85.7% vs. 56.7%; *p* < 0.001). Furthermore, the presence of a mediastinal cavity, a feature of more complex and challenging leaks, was significantly less frequent in the pEVT group (51.7% vs. 73.2%; *p* = 0.001). Sponge-positioning strategies reflected these differences. In the pEVT group, endoluminal sponge placement alone was sufficient in the majority of cases (56.7% vs. 32.1%; *p* < 0.001). In contrast, the non-pEVT group more frequently required an intracavitary approach (67.9% vs. 43.3%; OR, 0.37; 95% CI 0.16–0.82; *p* < 0.001). The duration of EVT was shorter in the pEVT group, with a median of 21 days compared with 26 days in the non-pEVT group. Similarly, the number of changes in EVT cycles was lower in the pEVT group (4 vs. 5), although this difference were not statistically significant. Additional local therapies for anastomotic fistulas (e.g., barium sulfate instillation using a contrast catheter through the endoscope to inject 1–2 ml of barium sulfate directly into the EVT conditioned the fistulous tract to promote local granulation or fibrin glue) were used at similar rates (see Table 2 for further details).

**Table 2:** Baseline therapy characteristics

	Total (*n* = 116) *n* (%)	Non-preemptive EVT (*n* = 56) *n* (%)	Preemptive EVT (*n* = 60) *n* (%)	*p* value
Leukocytes: 10^3^/mL (range)				
Day 3	8.7 (7.2–11)	9.4 (7.3–12.3)	8.4 (7.0–9.9)	Ns
Day 5	7.9 (6.6–9.9)	8.0 (6.7–10.9)	7.8 (6.4–9.2)	Ns
Day 10	11.4 (8.7–15.5)	13.0 (9.4–16.8)	10.1 (7.7–14.3)	**0.009**
C-reactive protein concentration: mg/dL (range)				
Day 3	18.2 (13.6–24.3)	20.2 (16.5–24.9)	15.8 (11.3–23.1)	**0.004**
Day 5	14.0 (8.7–20.9)	16.9 (10–25.1)	12.7 (7.3–17.6)	**0.022**
Day 10	17.6 (11.1–21.4)	19.0 (14.9–22.2)	14.2 (7.7–19.8)	**0.002**
Day AL was detected (range)	8 (6–12)	8 (6–12)	8 (6–11)	Ns
AL detected because of				
Scheduled endoscopy checkup	50 (43.1)	16 (28.6)	34 (56.7)	**0.011**
Elevated inflammation parameters	44 (37.9)	25 (44.6)	19 (31.7)	
Change in drain	10 (8.6)	8 (14.3)	2 (3.3)	
Clinical signs	12 (10.3)	7 (12.5)	5 (8.3)	
Anastomosis distance from incisors: cm (range)	28 (27–30)	28 (26–30)	28 (28–30)	Ns
Size of AL (fraction of circumference)				
< 10%	42 (36.2)	17 (30.4)	25 (41.7)	Ns
< 25%	35 (30.2)	15 (26.8)	20 (33.3)	
< 50%	33 (28.4)	21 (37.5)	12 (20)	
> 50%	6 (5.2)	3 (5.4)	3 (5)	
Mediastinal leakage cavity	72 (62.1)	41 (73.2)	31 (51.7)	**0.001**
Size of mediastinal leakage cavity: cm (range)	5 (3–10)	5 (31–10)	5 (2–7)	Ns
Localization of AL				
Left	60 (51.7)	26 (46.4)	34 (56.7)	Ns
Right	22 (19)	14 (25)	8 (13.3)	
Dorsal-left	12 (10.3)	8 (14.3)	4 (6.7)	
Dorsal-right	3 (2.6)	0 (0)	3 (5)	
Dorsal	19 (16.4)	8 (14.3)	11 (18.3)	
ZACC score				
2	25 (21.6)	3 (5.4)	22 (36.7)	**<0.001**
3	9 (7.8)	5 (8.9)	4 (6.7)	
4	82 (70.7)	48 (85.7)	34 (56.7)	
EVT positioning				
Endoluminal	52 (44.8)	18 (32.1)	34 (56.7)	**<0.001**
Intracavitary	64 (55.2)	38 (67.9)	26 (43.3)	
Duration of EVT: days (range)	24 (17–34)	26 (15–35)	21 (18–33)	Ns
EVT re-application: count (range)	4 (3–7)	5 (2–7)	4 (3–6)	Ns
Suction min or max: mmHg (range)				
Endoluminal, min	125 (125–140)	140 (125–150)	125 (125–140)	**0.035**
Endoluminal, max	150 (140–160)	150 (125–160)	150 (140–160)	Ns
Intracavitary, min	125 (100–125)	125 (125–125)	100 (50–125)	**0.001**
Intracavitary, max	135 (125–150)	140 (125–150)	130 (100–140)	Ns
Additional therapy				
Barium	50 (43.1)	22 (39.3)	28 (46.7)	Ns
Fibrin glue	10 (8.6)	4 (7.1)	6 (10)	
Therapy of non-healed AL				
Non-healed patients	20 (17.2)	11 (19.6)	9 (15)	Ns
OTSC	1 (5)	1 (9.1)	0 (0)	
Stent	1 (5)	1 (9.1)	0 (0)	
Re-anastomosis	1 (5)	1 (9.1)	0 (0)	
Cervical esophageal diversion	5 (25)	1 (9.1)	4 (44.4)	
Deceased	12 (60)	7 (63.6)	5 (55.6)	

Values in bold indicate statistical significance (*p* < 0.05)

EVT, endoscopic vacuum therapy;* n*, no. of patients; ns, nonsignificant; AL, anastomotic leakage; ZACC, Zurich Anastomotic Condition Classification; OTSC, over-the-scope clip

### Patient Outcomes

The rate of pulmonary complications (pneumonia, pleural effusion requiring treatment, or empyema) did not differ between the two groups. Overall healing rates were high and comparable between the groups (85% for pEVT vs. 80.4% for non-pEVT; *p* = 0.677). In cases for which endoscopic therapy failed, clinical outcomes were markedly worse. A substantial proportion of the 20 patients with persistent leaks required major salvage procedures such as cervical esophageal diversion (25%), and a total of 12 patients died (60%). No significant differences were observed in 30- and 90-day survival rates between the two groups. The non-pEVT group had longer overall stays in the ICU (21.5 vs. 8 days; *p* < 0.001) and hospital (51 vs. 34 days; *p* < 0.001) (see Table 3 for details).

**Table 3:** Patient outcomes

	Total (*n* = 116) *n* (%)	Non-preemptive EVT (*n* = 56) *n* (%)	Preemptive EVT (*n* = 60) *n* (%)	*p* value
yT				
In situ	1 (0.9)	1 (1.8)	0 (0)	Ns
0	17 (14.7)	6 (10.7)	11 (18.3)	
1	33 (28.4)	11 (19.6)	22 (36.7)	
2	21 (18.1)	9 (16.1)	12 (20)	
3	40 (34.5)	25 (44.6)	15 (25)	
4	2 (1.7)	2 (3.6)	0 (0)	
X	1 (0.9)	1 (1.8)	0 (0)	
Other	1 (0.9)	1 (1.8)	0 (0)	
yN				
0	60 (51.7)	27 (48.2)	33 (55)	Ns
1	24 (20.7)	13 (23.2)	11 (18.3)	
2	19 (16.4)	10 (17.9)	9 (15)	
3	13 (11.2)	6 (10.7)	7 (11.7)	
R				
R0	109 (94)	49 (87.5)	60 (100)	**0.005**
R1		7 (12.5)	0 (0)	
Pulmonary complications				
Pneumonia	37 (31.9)	20 (35.7)	17 (28.3)	Ns
Pleural effusion requiring treatment or empyema	49 (42.2)	25 (44.6)	24 (40)	
Healing of AL	96 (82.8)	45 (80.4)	51 (85)	Ns
Length of primary inpatient stay: days (range)	41 (29–57)	51 (35–76)	34 (25–47)	**<0.001**
Length of ICU stay: days (range)	16 (8–34)	21.5 (14–43)	8 (2–23)	**<0.001**
30-day survival	113 (97.4)	54 (96.4)	59 (98.3)	Ns
90-day survival	99 (86.1)	47 (83.9)	52 (88.1)	Ns

Values in bold indicate statistical significance (*p* < 0.05)

EVT, endoscopic vacuum therapy; *n*, number of patients; ns, nonsignificant; AL, anastomotic leakage; ICU, intensive care unit

### Multivariable Regression Analysis

Table 4 presents the results of the regression analyses assessing the impact of pEVT on several outcomes: ZACC score (grade II vs. grade III or grade IV), the need for intracavitary EVT, and the overall duration of hospital and ICU stays. The patients who received pEVT had 14.41 times higher odds of achieving a favorable ZACC score than those without pEVT (OR, 14.41; 95% CI 2.9–117.46; *p* = 0.004). Furthermore, this study population had significantly lower odds of requiring intracavitary vacuum therapy than the patients without pEVT. The odds ratio was 0.31 (95% CI 0.11–0.86; *p* = 0.027), indicating a 69% reduction in the odds of needing therapeutic intracavitary sponge placement when EVT was used prophylactically. Additionally, pEVT was associated with a significant reduction in both ICU and overall hospital stays. Converted to the original (untransformed) scale, pEVT reduced ICU length of stay by 74.4% (95% CI −84.7–−57.3%; *p* < 0.001), and hospital length of stay by 27.5% (95% CI −43.4–−7.2%; *p* < 0.011). Other clinical and demographic variables showed no significant association with the likelihood of achieving a ZACC score of 2, the need for intracavitary EVT, or the duration of hospital or ICU stay.

**Table 4:** Multivariable regression^a^

Variable	ZACC		Intracavitary vacuum therapy		Hospital length of stay		ICU length of stay	
	OR (95% CI)	*p* value	OR (95% CI)	*p* value	Slope (95% CI)	*p* value	Slope (95% CI)	*p* value
pEVT	**14.41 (2.9 to 117.46)**	**0.004**	**0.31 (0.11 to 0.86)**	**0.027**	**–0.32 (–0.57 to –0.07)**	**0.011**	**–1.36 (–1.88 to –0.85)**	**<0.001**
Age	1.04 (0.98 to 1.11)	0.258	0.99 (0.94 to 1.03)	0.525	0.01 (0 to 0.02)	0.192	0.01 (–0.01 to 0.03)	0.446
Sex	7.15 (0.98 to 63.08)	0.056	0.34 (0.08 to 1.32)	0.121	–0.24 (–0.57 to 0.08)	0.144	–0.52 (–1.2 to 0.16)	0.134
ASA ≥3	0.85 (0.28 to 2.52)	0.775	0.77 (0.33 to 1.79)	0.545	–0.21 (–0.41 to –0.01)	0.039	–0.4 (–0.81 to –0.02)	0.064
BMI	0.94 (0.84 to 1.06)	0.324	0.98 (0.89 to 1.07)	0.618	0 (–0.02 to 0.02)	0.860	–0.02 (–0.06 to –0.02)	0.373
Diabetes mellitus	2.69 (0.25 to 23.17)	0.372	2.67 (0.57 to 15.62)	0.235	0.11 (–0.25 to 0.47)	0.540	0.07 (–0.68 to 0.82)	0.846
Cardiovascular comorbidity	0.65 (0.17 to 2.67)	0.529	0.39 (0.12 to 1.16)	0.101	–0.21 (–0.47 to 0.05)	0.107	–0.26 (–0.8 to 0.28)	0.343
Neoadjuvant therapy	0.51 (0.13 to 2.06)	0.334	2.23 (0.69 to 7.65)	0.184	0 (–0.28 to 0.28)	0.985	–0.33 (–0.91 to 0.25)	0.262
Feeding tube	0.9 (0.18 to 3.98)	0.891	0.81 (0.23 to 2.8)	0.734	–0.14 (–0.44 to 0.16)	0.368	–0.5 (–1.12 to 0.13)	0.118
Duration of surgery	1 (1 to 1.01)	0.200	1 (1 to 1.01)	0.596	0 (0 to 0)	0.920	0.11 (–0.03 to 0.25)	0.126
Distance of anastomosis from incisors	1.04 (0.83 to 1.28)	0.738	0.88 (0.75 to 1.01)	0.087	–0.01 (–0.05 to 0.02)	0.501	–0.01 (–0.08 to 0.07)	0.884

Values in bold indicate statistical significance (*p* < 0.05)

*ZACC, Zurich Anastomotic Condition Classification; OR, odds ratio; CI, confidence interval; ICU, intensive care unit; pEVT, preemptive endoscopic vacuum therapy; ASA, American Society of Anesthesiologists; BMI, body mass index*

^a^Results of the regression analyses assessing the impact of pEVT on ZACC score (grade II vs. grade III or IV), the need for intracavitary vacuum therapy (endoluminal vs. intracavitary), and the overall duration of hospital and ICU stays. The reported results for hospital and ICU lengths of stay are presented on the transformed (logarithmic) scale

## Discussion

This study investigated the impact of pEVT on clinical outcomes for esophageal cancer patients who experienced AL after Ivor Lewis esophagectomy, comparing those treated with and without pEVT. Both groups achieved similarly high AL healing rates (85% for pEVT vs. 80.4% for non-pEVT) through endoscopic management. However, pEVT was associated with less severe leaks, reduced need for complex interventions, and less severe mediastinitis and inflammation, resulting in significantly shorter ICU and hospital stays. To the best of our knowledge, this is the first larger case series directly comparing outcomes of AL with and without pEVT. The findings are based on a cohort that reflected a typical Western esophageal cancer population.

### pEVT as a Modulator of Leak Severity and Postoperative Recovery

Although AL still occurred despite the use of pEVT, this approach was associated with a more favorable clinical course that remained manageable endoscopically. Leaks were more often identified during scheduled surveillance endoscopies rather than triggered by clinical deterioration of the patient, indicating that pEVT enables earlier and more controlled intervention. This was accompanied by a markedly milder inflammatory response in the early postoperative period, as indicated by lower systemic inflammatory markers. It is well established in the literature that early detection of AL is crucial because leak severity is closely linked to the extent of contamination and systemic inflammation, both of which increase with delayed diagnosis.^15,16^ Therefore, the lower inflammatory markers observed in this study are important because they reflect a reduced inflammatory insult.

Moreover, pEVT significantly reduced leak severity, as reflected by lower ZACC scores, and was independently associated with 14-fold increased odds of favorable ZACC grading in multivariable regression analysis. This was further supported by a 69% reduction in the odds of requiring intracavitary sponge therapy, which typically is reserved for managing more severe or complex leaks. Therefore, pEVT may help prevent clinically significant or high-grade anastomotic leaks.

Our findings are consistent with emerging evidence suggesting that pEVT, although not preventing anastomotic leaks entirely, can significantly mitigate their clinical severity. As reported by Neumann et al.^17^, Gubler et al.^18^, and Müller et al.^6^, early endoscopic stabilization via pEVT appears to improve local healing conditions and reduce the risk of leak progression.

The rationale for pEVT builds on its proven effectiveness in treating established leaks and the growing consensus that early preemptive intervention may prevent more severe complications. By potentially preventing clinical deterioration and enabling timely intervention, pEVT also may contribute to reducing failure-to-rescue rates for patients with AL. Neumann et al.^17^ observed that pEVT halted progression in patients with suspected mucosal ischemia,^17^ whereas Gubler et al.^18^ reported a moderate reduction in leak incidence and postoperative morbidity.^18^ Müller et al.^6^ further substantiated these benefits in a larger cohort (*n* = 67), introducing both a risk-adapted pEVT strategy and the ZACC classification, tools that have since been applied in our own study to enhance consistency in assessment and outcome reporting.^6^ Even in high-risk scenarios, such as redo anastomoses, recent data by Chon et al.^19^ point toward potential benefits of pEVT.

Although all studies were retrospective and lacked control groups, the consistently low leak-related morbidity across cohorts supports the notion that pEVT offers clinical value, particularly for selected patient populations.

Although pEVT effectively reduced leak severity, this improvement did not translate into a reduction of pulmonary complications, which remained equally frequent in both groups, reflecting the well-established link between AL and secondary pulmonary morbidity. Compared with open esophagectomy, RAMIE has been associated with fewer pulmonary complications,^20^ but these findings were reported in unselected patient cohorts. In our study population, which exclusively included patients with AL, pulmonary complication rates were comparable between groups, indicating that the pulmonary benefits of RAMIE described in unselected cohorts may not extend to this high-risk subgroup.

The positive findings regarding improved management of AL are not merely of clinical interest. They also translate into tangible improvements in postoperative recovery. Most notably, the ICU stay was reduced by 74.4%, and the overall hospital length of stay was reduced by 27.5% in the pEVT group. In esophageal cancer surgery, ICU stays are a major cost driver, particularly when prolonged by complications such as severe AL. As we demonstrated earlier, the ICU length of stay is among the most decisive factors contributing to the economic burden of esophagectomy.^4^ These complications not only increase direct health care expenditures, but also strain hospital infrastructure and staff resources. In this context, the ability of pEVT to reduce leak severity, and thus contribute to limiting the need for prolonged ICU treatment, may offer both clinical and economic value. Whereas overall improvements in surgical technique and perioperative care during the study period also contributed to shorter hospital stays, pEVT likely played a targeted role in mitigating the consequences of anastomotic complications.

### EVT Management for AL: Gaps in Evidence and Contributions from Our Findings

Currently, detailed information in the literature on the ideal management of EVT for AL is limited. In particular, standardized protocols regarding optimal treatment duration and negative pressure settings are lacking. Our data offer valuable insights into the effective application of pEVT directly at the anastomosis and tEVT with higher negative pressure settings including endoluminal (median maximum, −150 mmHg) and intracavitary (median maximum, −135 mmHg). The EVT was performed under a restricted sip-drinking protocol, limited to 200 mL per day. Additional therapies, such as barium, may further support complete healing down to the mucosal level of the AL.

### Balancing Benefits and Burdens: Patient Selection for pEVT

A major drawback of pEVT is the necessary postoperative fasting, which poses a significant challenge for already vulnerable patients. This nutritional restriction delays recovery, requires parenteral or jejunal feeding, and conflicts with enhanced recovery protocols emphasizing early oral intake and mobilization. Consequently, applying pEVT universally may result in overtreatment. Therefore, pEVT is best reserved for high-risk patients, as defined by risk stratification models. In the future, preemptive treatment pathways could be expanded through precise patient selection using, for example, artificial intelligence for risk assessment or via bioactive EVT materials. This targeted approach aims to maximize therapeutic benefit while minimizing unnecessary nutritional compromise and procedural burden. Thus, pEVT should be seen as a promising adjunct in modern esophageal surgery, with potential to enhance recovery and reduce resource-intensive complications such as prolonged ICU stays for high-risk patients. Data from prospective trials such as the ongoing preSPONGE study^21^ are needed to validate these results and refine patient selection criteria.

## Study Limitations

The main limitations of this study relate to its retrospective, single-center design and lack of randomization. Temporal changes in surgical technique, particularly the transition from open esophagectomy to RAMIE, raise the concern that differences between the pEVT and non-pEVT groups might be confounded by the surgical approach. Although meta-analyses suggest that access type does not significantly influence leak rates^20^ and may even favor conventional MIE over RAMIE,^22^ this concern should be interpreted with caution.

In our cohort, more stapled anastomoses were performed in the pEVT group due to the transition to RAMIE. Because a leak after a stapled anastomosis is generally smaller than after a hand-sewn anastomosis, this factor may have contributed to differences in leak size and severity. Nevertheless, because our study focused on AL management rather than incidence, we believe the overall impact of the surgical approach on the main findings was limited. All stapled anastomoses were performed using a standardized technique. The RAMIE procedure typically is associated with longer operative times^20^ (a finding also seen in our cohort), which correlate with higher complication rates and less favorable outcomes.^23,24^ Moreover, RAMIE implementation follows a known learning curve.^25^ Nevertheless, we did not observe increased leak severity or treatment complexity during this early phase. On the contrary, even with these factors, the patients in the pEVT group showed a more favorable clinical course.

Beyond surgical technique, the long study period included changes in perioperative oncologic protocols. Most patients received neoadjuvant therapy, primarily radiochemotherapy per CROSS (72%). Although current standards have evolved (e.g., adjuvant nivolumab [CheckMate 577^26^] and perioperative FLOT [ESOPEC^27^]), neoadjuvant treatment has not been shown to significantly affect AL rates.^28,29^ Therefore, despite these developments, we believe our findings remain relevant to current clinical practice.

## Conclusion

This study, based on one of the largest cohorts of patients with AL after esophagectomy and the first to directly compare pEVT with non-pEVT management, provides important evidence that pEVT may positively influence the clinical course of AL. Our findings suggest that pEVT is associated with reduced leak severity, lower incidence of mediastinitis, decreased postoperative inflammation, and shorter ICU and hospital stays, indicating a potential benefit for selected high-risk patients. Future multicenter studies are needed to confirm these findings and further define standardized indications for the optimal use of pEVT in clinical practice.

## Data Availability

All data generated and/or analyzed during this study are included in this published article.
